# The blood pressure sensitivity to changes in sodium intake is similar in Asians, Blacks and Whites. An analysis of 92 randomized controlled trials

**DOI:** 10.3389/fphys.2015.00157

**Published:** 2015-05-21

**Authors:** Niels Graudal, Gesche Jürgens

**Affiliations:** ^1^Department of Rheumatology, University Hospital of CopenhagenCopenhagen, Denmark; ^2^Unit of Clinical Pharmacology, Roskilde HospitalRoskilde, Denmark

**Keywords:** blood pressure, dietary sodium, dietary salt, ethnicity, meta-analysis

## Abstract

The purpose of the meta-analysis of randomized trials was to analyze the significance of ethnicity on the effect of sodium reduction (SR) on blood pressure (BP) by estimating the effect of SR on BP in Asians, Blacks and Whites under conditions, which were adjusted with respect to baseline BP, baseline sodium intake and quantity of SR. Relevant studies were retrieved from a pool of 167 RCTs published in the period 1973–2010 and identified in a previous Cochrane review. 9 Asian, 9 Black, and 74 White populations standardized with respect to the range of baseline blood pressure, the range of baseline sodium, duration of SR (at least 7 days) and baseline sodium intake (at maximum 250 mmol) intake were included. In the cross-sectional analysis, there was no difference in change in SBP to SR between the ethnic groups, but there was a small difference in SR induced change in DBP between Blacks and Whites (*p* = 0.04). The comparison of changes in SBP and DBP to SR in ethnic groups compared in identical studies showed no statistically significant differences between the groups.

## Introduction

The effect of sodium reduction (SR) on blood pressure (BP) is smaller in persons with normal blood pressure than in hypertensive persons (Graudal et al., 1998, 2011). A recent dose-response analysis showed a dose-response relationship between SR and effect on BP in study populations with a mean BP above 130/80 mmHg, but not in study populations with a mean BP below 130/80 mmHg, unless SR was applied on study populations with an extreme sodium intake above about 6 g sodium (14.5 g salt) (Graudal et al., 2015). These findings indicate that the effect of SR depends on the baseline blood pressure, the baseline sodium intake and the quantity of SR. Hypothetically, the duration of SR could also be an important determinant of the effect of SR on BP, but a detailed analysis of 15 longitudinal RCTs, which investigated participants repeatedly during up to 6 weeks, showed that the effect of SR on BP was similar during the observation period from 1 to 6 weeks indicating that the duration of SR beyond 1 week does not influence the effect of SR on BP (Graudal et al., 2015).

Our previous meta-analysis showed that the effect of SR on SBP was higher in Asian hypertensive persons (10.2 mmHg) than in White (5.5 mmHg) and Black hypertensive persons (6.4 mmHg) (Graudal et al., 1998). Furthermore the effect of SR on SBP was higher in Black persons with a normal blood pressure (4.0 mmHg) than in Asian (1.3 mmHg) and White persons with a normal BP (1.3 mmHg) (Graudal et al., 1998). However, a subsequent analysis of Blacks showed that the difference between Blacks and Whites was small, if studies investigating extreme sodium reductions were eliminated (Graudal and Alderman, 2014). The reason for ethnic differences are not defined and could be due to different baseline blood pressures, sodium intakes and doses of SR or other confounders, rather than genetic differences.

The purpose of the present supplementary analysis of our previous meta-analysis of randomized trials (Graudal et al., 2011) was to analyze the significance of ethnicity on the effect of SR on BP by estimating the effect of SR on BP in Asians, Blacks and Whites under conditions, which were adjusted with respect to baseline BP, baseline sodium intake and quantity of SR.

## Material

Relevant studies were retrieved from a pool of 167 RCTs published in the period 1973–2010 and identified in a Cochrane review in 2011 (Graudal et al., 2011). Search methods for identification of these 167 studies are previously described in detail (Graudal et al., 2011).

## Methods

### Eligibility criteria

Trials randomizing participants to two different sodium intakes were included provided that the sodium intake was measured as 24-h urinary excretion (Graudal et al., 2011). Baseline BP was used to define the study groups to ensure that the baseline BP was similar across the three ethnic study groups. The most extreme sodium intake in the world's populations is about 6 g sodium (14.5 g salt) (McCarron et al., 2013; Powles et al., 2013), corresponding to about 250 mmol, and we therefore excluded studies with sodium intakes above this level, as such intakes reflected an experimental situation rather than a population norm. In a series of longitudinal RCTs we have showed that the effect of SR on BP is at maximum after 1 week (Graudal et al., 2015). As we do not know whether this effect is reached before 1 week, we excluded studies with a shorter duration than 1 week.

In order to avoid excluding the relatively few studies, which have been performed in Blacks and Asians, the baseline systolic BP (SBP) range of these study groups were used to define the baseline SBP range of the white comparator study groups.

### Bias

The following factors were analyzed across the defined ethnic groups to evaluate the comparability of the groups: Age, baseline SBP, baseline diastolic BP (DBP), baseline sodium intake and the quantity of sodium reduction.

### Statistical analysis

In this re-analysis featuring improved control for blood pressure and the extent of salt intake and salt reduction the individual study data were integrated in meta-analyses separately for Asians, Blacks, and Whites, and the integrated summary data for each of the groups were then compared versus each other. As well the separate meta-analyses as the comparisons of the summary data for each ethnic group were performed by means of the inverse variance method (continuous data) in Review Manager (RevMan) [Computer program], version 5.1. Copenhagen: The Nordic Cochrane Centre, the Cochrane Collaboration, 2008.

A supplementary meta-regression analysis of Ethnicity versus effect on blood pressure (separate analyses for SBP and DBP) adjusted for amount of sodium reduction, baseline blood pressure (SBP and DBP), age, and the duration of the sodium reduction intervention was performed by means of the multiple regression analysis package in Statview 5.0.

## Results

### Study selection

The study groups were defined from 167 references (Graudal et al., 2011). Some references reported separate data on sodium sensitive and sodium resistant participants. These were integrated before inclusion in the meta-analysis. Some references included separate analyses on hypertensive and normotensive persons or on different ethnic groups. These were included as separate data. The total number of study groups in the 167 references (Graudal et al., 2011) was 184. 16 study groups of mixed ethnic populations were excluded. Two Black populations with duration of sodium reduction less than 7 days were excluded. As it was the objective to match studies for each ethnic group according to range of SBP, all Asian and Black populations were ranked with respect to baseline SBP. Ten Asian study populations had a mean baseline SBP in the range 113–158 mm Hg and 12 Black populations had mean baseline SBP in the range of 108–156 mmHg. We excluded the one study with the lowest baseline SBP of 108 mmHg, as this was outside the 113–158 range defined by the Asian populations. In this study SBP raised 0.5 mmHg when sodium intake was reduced. The remaining 11 studies had a baseline SBP range of 113–156 mmHg and thus were comparable to the Asian population concerning this range. As SBP in the 144 studies of white populations varied in the range of 105–179 mmHg, 15 with baseline SBP above 158 mmhg and 18 with a baseline SBP in the range of 105–112 mmHg were excluded. Five white study populations with no information on baseline SBP were also excluded, leaving 106 study populations with a baseline SBP in the range of 113–158. Then 1 study of Asians, 2 studies of blacks and 21 studies of whites with a high sodium intake above 250 mmol and 11 white populations with duration of SR less than 7 days were excluded. Thus, 9 Asian populations (6 references, Table 1), 9 black populations (7 references, Table 1) and 74 white populations (70 references, Table 1) standardized with respect to the range of baseline blood pressure, duration of SR (at least 7 days) and baseline sodium intake (at maximum 250 mmol) were included.

**Table 1:** **Baseline data, blood pressure outcome data and references of included studies**.

**Eth**.	**Authors**	**Dur**.	**BP**	**N**	**Age**	**HS**	**LS**	**B SBP**	**B DBP**	**E SBP**	**SSE**	**E DBP**	**DSE**	**References**
A	Ishimitsu	7	N	7	53	217	22	116	77	−2.0	4.3	−2.0	3.6	Clin. Sci. 1996; 91, 293–298.
A	Ishimitsu	7	H	23	55	217	24	157	95	−15.7	6.0	−5.5	3.2	Clin. Sci. 1996; 91, 293–298.
A	Uzu	7	H	70	51	204	31	154	94	−15.6	7.4	−5.0	3.1	AJH 1999; 12, 35–39.
A	Suzuki	7	H	20	59	167	51	157	92	−4.0	2.0	−2.6	1.3	Hypertension 2000; 35, 864–868.
A	Nakamura	42	N	38	47	240	216	113	66	2.0	2.0	−5.5	3.0	Circ. J. 2003;67, 530–534.
A	Nakamura	42	TH	26	47	240	216	140	90	−5.8	4.9	−1.3	3.3	Circ. J. 2003;67, 530–534.
A	Takahashi	365	N	341	56	248	209	123	74	−2.3	1.2	−1.2	1.0	J. Hypertens. 2006; 24, 451–8.
A	Takahashi	365	TH	107	56	248	209	143	83	−5.2	2.4	0.1	1.7	J. Hypertens. 2006; 24, 451–458.
A	He	42	H	29	47	176	108	142	92	−5.4	1.9	−2.2	1.0	Hypertension 2009; 54, 482–488.
B	Dubbert	90	TH	67	61	194	160	138	86	−1.4	3.8	−0.5	1.7	Behav. Ther. 1995; 26, 721–732.
B	Sacks	30	N	68	48	141	64	129	84	−6.4	1.2	−4.0	0.8	NEJM 2001; 344, 3–10.
B	Sacks	30	H	46	48	141	64	143	89	−8.6	1.2	−5.3	0.8	NEJM 2001;344:3-10.
B	Appel	105	TH	142	66	145	116	128	71	−5.0	1.7	−2.9	1.2	Arch. IM 2001; 161, 685–693.
B	Palacios	21	N	15	12	109	35	113	59	3.4	1.5	−0.1	1.9	JCEM 2004; 89, 1858–1863.
B	Forrester (Ni)	21	N	58	47	127	53	115	73	−4.8	1.5	−3.2	1.0	J. Hum. Hypertens. 2005; 19, 55–60.
B	Forrester (Ja)	21	N	56	41	155	68	126	76	−5.1	1.5	−2.2	1.5	J. Hum. Hypertens. 2005; 19, 55–60.
B	Swift	28	H	40	50	167	89	156	100	−8.0	2.1	−3.0	1.1	Hypertension 2005; 46, 308–312.
B	He	42	H	69	50	165	121	149	90	−4.8	1.2	−2.2	0.7	Hypertension 2009; 54, 482–488.
W	Skrabal	14	N	20	23	200	50	125	73	−2.7	2.1	−3.0	1.5	Lancet 1981; II, 895–900.
W	Ambrosioni	42	H	25	23	120	60	130	75	−2.2	1.6	−0.4	1.2	Hypertension 1982; 4, 789–794.
W	Beard	84	TH	90	49	161	37	141	87	−5.2	4.9	−3.4	2.9	Lancet. 1982; II, 455–458.
W	Puska	72	N	38	40	167	77	131	82	−1.5	4.5	−2.1	2.8	Lancet 1983; I, 1–5.
W	Puska H	72	H	34	40	167	77	147	98	1.8	5.6	0.5	3.1	Lancet 1983; I, 1–5.
W	Watt	28	H	18	52	143	87	150	91	−0.5	1.5	−0.3	0.8	BMJ 1983; 286, 432–6.
W	Skrabal	14	N	52	23	194	38	121	64	−3.1	4.4	−1.9	2.6	Hypertension 1984; 6, 152–158.
W	Fagerberg	63	H	30	51	195	96	149	98	−3.7	7.1	−3.1	4.1	BMJ 1984; 288, 11–14.
W	Maxwell	84	H	30	47	200	39	148	98	−2.0	6.7	2.0	3.8	Arch. IM 1984; 144, 1581–1584.
W	Richards	28	H	12	36	180	80	137	86	−4.0	2.8	−3.0	2.3	Lancet 1984; I, 757–761.
W	Tuthill	56	N	191	16	126	65	113	70	0.0	1.1	0.0	1.3	Tox. Ind. Health 1985; 1, 35–43.
W	Skrabal	14	N	62	23	194	40	120	64	−3.1	2.2	−1.5	0.9	SJCLI 1985; 176(S), 47–57.
W	Teow	14	N	9	25	240	40	114	66	−0.6	1.2	−2.7	1.4	Clin. Exp. Hypertens. 1986; A7, 1681–1695.
W	ANHMRC	84	H	100	53	150	80	150	94	−4.8	3.9	−4.2	1.9	J. Hypertens. Suppl. 1986; 4, S629-S637.
W	Fuchs	9	N	17	20	241	12	117	57	−3.6	2.2	1.9	1.0	Braz. J. Med. Biol. Res. 1987; 20, 25–34.
W	Morgan	60	TH	20	61	135	78	143	82	−6.0	9.0	−4.0	4.3	Lancet 1987; I, 227–230.
W	Grobee	42	H	40	24	129	57	143	78	−0.8	1.5	−0.8	1.4	BMJ 1987; 293, 27–29.
W	McGregor	30	TH	15	52	183	83	150	97	−13.0	3.3	−9.0	3.1	BMJ 1987; 294, 531–534.
W	Morgan	14	H	8	63	135	68	149	96	−7.0	3.0	−6.0	3.0	J. Hypertens. 1988; 6(Suppl. 4), S652–S654.
W	Sudhir	12	N	6	35	163	29	129	81	−7.9	3.4	−5.0	2.1	Clin. Sci. 1989; 77, 605–610.
W	Hargreaves	14	N	8	23	155	49	129	66	−6.0	2.2	−3.0	2.0	Clin. Sci. 1989; 76, 553–557.
W	ANHMRC	48	H	103	58	153	90	154	95	−5.5	1.5	−2.8	0.8	Lancet 1989; 1, 399–402.
W	Schmid	7	N	9	32	210	20	125	75	−3.0	1.9	3.0	1.6	J. Hypertens. 1990; 8, 277–283.
W	Schmid H	7	H	9	36	210	29	147	93	−6.0	3.1	−1.9	2.1	J. Hypertens. 1990; 8, 277–283.
W	Sharma	7	N	40	25	239	25	113	71	−2.1	1.1	−3.1	1.0	Hypertension 1990; 16, 407–413.
W	Friberg	13	N	10	33	152	35	114	69	0.0	2.0	−1.0	2.0	Hypertension 1990; 16, 121–130.
W	Del Rio	14	H	15	49	190	90	149	94	−3.4	2.0	−1.1	1.8	Rev. Clin. Esp. 1990; 186, 5–10.
W	Parker	28	TH	59	52	142	69	138	85	1.3	2.2	0.6	0.9	Hypertension 1990; 16, 398–406.
W	Howe	28	N	90	13	179	98	115	60	−1.0	0.7	−0.6	0.7	J. Hypertens. 1991; 9, 181–186.
W	Mascioli	28	N	48	52	179	109	131	84	−3.6	0.9	−2.3	0.8	Hypertension 1991; 17(Suppl. 1), I21–I26.
W	Egan H	7	H	18	35	214	20	124	78	−2.7	5.5	−1.7	3.5	AJH 1991; 4, 416–421.
W	Gow	7	N	9	0	111	17	120	68	−8.0	1.6	−3.0	2.2	EJCP 1992; 43, 635–638.
W	Cobiac	28	N	106	67	148	75	132	77	−2.8	1.6	−1.0	1.8	J. Hypertens 1992; 10, 87–92.
W	Benetos	28	H	20	42	163	85	149	93	−6.5	1.9	−3.7	1.3	J. Hypertens, 1992; 10, 355–360.
W	Sciarrone	56	TH	91	54	134	52	136	83	−5.8	4.1	−0.4	2.3	J. Hypertens 1992; 10, 287–298.
W	Nestel	42	N	66	66	157	91	125	73	−3.2	2.7	−1.4	2.0	J. Hypertens 1993; 11, 1387–1394.
W	Del Rio	14	H	30	49	199	48	156	96	−1.4	1.8	−0.5	1.3	JIM 1993; 233, 409–414.
W	Zoccali	7	H	15	45	217	54	144	92	−14.0	2.5	−8.0	1.4	J. Hypertens. 1994; 12, 1249–1253.
W	Jula	365	H	76	44	166	109	147	97	−6.7	3.9	−3.8	1.7	Circulation 1994; 89, 1023–1031.
W	Howe	42	TH	56	55	158	78	145	81	−4.2	2.9	−1.5	1.9	J. Hum. Hyp. 1994; 8, 43–49.
W	Miller	14	N	36	23	191	133	118	62	1.9	1.6	−0.1	1.5	Psychosom. Med. 1995; 57, 381–389.
W	Fliser	7	N	7	26	203	23	114	71	−1.1	2.9	−0.7	1.8	EJCI 1995; 25, 39–43.
W	Arrol	182	TH	181	55	122	106	145	89	−0.4	3.4	−1.2	2.1	NZ Med. J. 1995; 108, 266–268.
W	Dubbert	90	TH	55	63	200	145	148	85	−1.4	3.8	−0.5	1.7	Behav. Ther. 1995; 26, 721–732.
W	Grey	7	N	34	23	185	52	116	70	1.0	1.2	1.0	0.9	AJH 1996; 9, 317–322.
W	Feldman H	7	H	8	27	182	6	126	79	2.6	2.9	1.6	1.8	CPT 1996; 60, 444–451.
W	Schorr	28	N	16	64	166	105	140	84	−1.0	2.7	0.0	1.7	J. Hypertens 1996; 14, 131–135.
W	Cappucio	30	N	18	67	167	91	149	85	−8.1	2.8	−3.9	1.5	Lancet 1997; 350, 850–854.
W	van Buul	196	N	232	28	140	75	122	71	0.0	1.8	0.0	1.2	Hyp. Preg. 1997; 16, 335–346.
W	Meland	56	H	16	50	191	125	146	95	−4.0	2.5	−3.0	1.4	SJCLI 1997; 57, 501–506.
W	Feldman	7	N	8	33	207	48	130	82	0.0	5.5	0.0	3.6	AJH 1999; 12, 643–647.
W	Barba	7	N	7	32	177	23	118	74	−3.2	5.5	−2.1	3.5	J. Hypertens. 2000; 18, 615–621.
W	Sacks	30	N	54	49	141	64	129	84	−4.0	1.2	−1.4	0.8	NEJM 2001; 344, 3–10. AIM 2001; 135, 1019–1028.
W	Sacks H	30	H	37	49	141	64	143	89	−6.6	1.2	−2.7	0.8	NEJM 2001; 344, 3-10. AIM 2001; 135, 1019–1028.
W	Seals	90	H	35	64	132	86	143	78	−8.0	2.6	−2.0	1.7	J. Am. Coll. Cardiol. 2001; 38, 506–513.
W	Appel TH	105	TH	471	66	145	105	128	71	−4.0	1.0	−1.6	0.7	Arch. IM 2001; 161, 685–693.
W	Johnson	14	H	40	69	185	112	150	83	−4.5	2.1	−0.6	1.5	J. Hypertens. 2001; 19, 1053–1060.
W	Manunta	14	H	20	48	177	67	152	99	−5.2	2.0	−3.3	2.0	Hypertension 2001; 38, 198–203.
W	Kleij	7	N	27	24	236	50	119	74	0.2	3.3	0.1	2.1	JASN 2002; 13, 1025–1033.
W	Kerstens	7	N	28	23	248	42	115	72	3.1	2.0	2.0	1.3	JCEM 2003; 88, 4180–4185.
W	Nowson	28	N	92	45	139	51	123	75	−0.7	0.8	0.0	0.6	J. Nutr. 2003; 133, 4118–4123.
W	Palacios	21	N	8	13	120	34	113	55	−0.1	1.5	4.2	1.7	JCEM 2004; 89, 1858–1863.
W	Gates	28	H	12	64	155	60	140	84	−3.0	1.8	−1.2	1.5	Hypertension 2004; 44, 35–41
W	Damgaard	7	N	12	57	188	59	124	77	0.0	4.7	0.0	3.0	AJP RICP 2006; 290, R1294–R1301.
W	Melander	28	M	39	53	140	51	144	91	−6.0	1.2	−2.3	0.9	J. Hypertens. 2007; 25, 619–627.
W	Dengel	8	H	28	63	191	36	152	79	−10.0	3.6	−4.0	3.6	Physiol. Res. 2007; 56, 393–401.
W	Jessani	7	N	184	50	138	57	122	79	−1.0	0.8	−1.0	0.8	AJH 2008; 21: 1238–1244.
W	Dickinson	14	N	29	53	156	64	116	73	−5.0	1.5	−1.0	1.1	AJCN 2009; 89, 485–490.
W	He	42	H	71	52	165	110	146	90	−4.8	1.2	−2.2	0.7	Hypertension 2009; 54, 482–488.
W	Meland	56	H	46	56	126	83	156	93	−5.0	3.8	−5.0	1.4	SJPHC 2009; 27, 97–103.
W	Nowson TH	98	TH	35	59	113	69	130	80	−5.5	2.7	−3.6	1.6	Nutr. Res. 2009; 29, 8–18.
W	Nowson	98	N	59	59	113	69	131	81	−1.1	2.0	0.3	1.5	Nutr. Res. 2009; 29, 8–18.
W	Weir	28	H	132	52	208	85	139	87	−9.4	1.0	−5.7	0.7	J. Cardiovasc. PT 2010; 15, 356–363.
W	Starmans-Kool	14	N	10	32	191	94	114	65	−2.0	3.4	0.0	3.4	J. Appl. Physiol. 2011; 110, 468–471.

*Eth, Ethnicity; A, Asian; B, Black; W, White; NI, Nigeria; Ja, Jamaica; ANHMRC, Australian National Health and Medical Research Council; Dur, Duration (Days); BP, Blood pressure; H, Hypertension; TH, Treated hypertension; N, Normotension; Age, Age (years); HS, High sodium intake (mmol); LS, Low sodium intake (mmol); B., Baseline; E, Effect; SBP, Systolic blood pressure; DBP, Diastolic blood pressure; SSE, Systolic blood pressure standard error; DSE, Diastolic blood pressure standard error*.

Table 1 shows the references, baseline characteristics and the individual study data of the 9 Asian, 9 Black, and 74 White populations included in the present study. Summary measures of baseline variables and BP effects of Asians, Blacks and Whites are shown in Table 2. There was no statistical difference in sex distribution although there was a trend toward a higher fraction of females in Asians. Asians were significantly older and had a significantly higher sodium intake than Blacks and Whites. Still the effect of SR on SBP and DBP did not differ from the effect in Blacks and Whites.

**Table 2:** **Baseline variables and blood pressure effects in populations of Whites, Blacks, and Asians balanced with respect to baseline blood pressure unadjusted and adjusted for sodium reduction**.

	**Asians (A)**	**Blacks (B)**	**Whites (W)**	W (A)^*^	**Significance**
N studies	9	9	74	39	
N participants	661	561	3782	2779	
Female %	58	45	45		0.21
	Mean (95% CI)				
Sodium reduction (SR) mmol	97.89 [46.46, 149.31]	63.16 [48.86, 77.46]	102.91 [92.20, 113.63]	66.12 [58.49, 73.75]	B vs. W 0.006
Age, years	52.30 [49.52, 55.08]	46.83 [25.41, 68.25]	43.04 [39.64, 46.44]	49.14 [43.57, 54.70]	A vs. W: 0.03
Baseline SBP, mm Hg	138.29 [126.71, 149.87]	132.84 [125.30, 140.37]	133.58 [130.75, 136.41]	137.24 [133.39, 141.09]	NS
Baseline DBP, mm Hg	84.68 [78.03, 91.33]	80.90 [75.01, 86.80]	80.69 [78.20, 83.18]	82.43 [79.04, 85.82]	NS
Baseline sodium intake	217.48 [194.68, 240.28]	148.70 [133.28, 164.12]	167.67 [161.17, 174.17]	137.43 [133.17, 141.68]	A vs. B and A vs. W: 0.00001
Effect SBP, mm Hg	−3.83 [−6.35, −1.31]	−4.68 [−7.10, −2.26]	−3.24 [−4.01, −2.46]	−3.06 [−3.94, −2.18]	NS (Figure 1)
Effect DBP, mm Hg	−1.99 [−3.04, −0.94]	−2.99 [−3.95, −2.02]	−1.54 [−2.05, −1.03]	−1.51 [−2.04, −0.98]	B vs. W: 0.04 B vs. W (A): 0.013 (Figure 1)

* *Sodium reduction adjusted to Blacks by elimination of all studies of Whites with SR > 90 mmol*.

**Figure 1:**
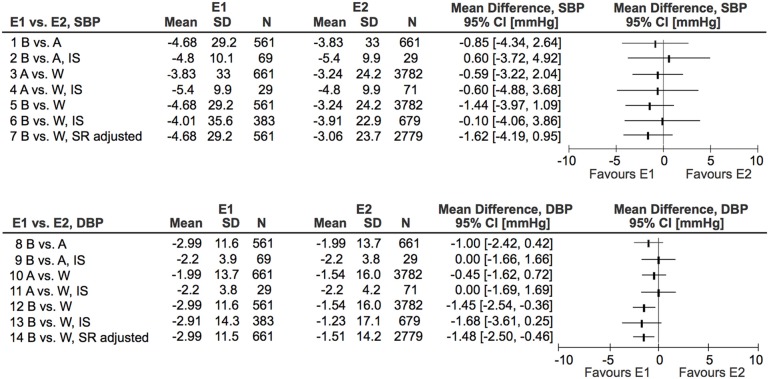
**Differences in effect of sodium reduction on systolic and diastolic blood pressure (SBP and DBP) between Asians (A), Blacks (B), and Whites (W)**. E, Ethnic group; IS, Identical study.

Figure 1 shows the comparisons between ethnic groups of the summary measures of SBP and DBP. Cross-sectional data for all included studies are shown in Figure 1, lines 1,3,5,8, 10, and 12. In addition the results from studies investigating at least two ethnic groups are shown in lines 2, 4, 6, 9, 11, and 13. The differences between the ethnic groups are substantially smaller than in the original analyses (Graudal et al., 2011). The effect of SR on DBP showed a significant difference between Blacks and Whites. The differences were generally smaller and not statistically significant when comparing the data obtained from studies investigating two or three ethnic groups in identical studies (Figure 1, lines 2, 4, 6, 9, 11, and 13) than when comparing data across studies (Figure 1, lines 1,3,5,8, 10, and 12).

### Supplementary analyzes

As the attempt to adjust the ethnic groups did not completely succeed, we supplied with a supplementary meta-regression analysis of ethnicity versus effect on blood pressure adjusted for amount of sodium reduction, baseline blood pressure, age, the duration of the sodium reduction intervention and the female percentage. This analysis shows that there is a statistically significant difference in SBP effect between the ethnic groups, both unadjusted and adjusted, but clinically the difference is small (about 1 mmHg) (Table 3). There were no differences in DBP effect between the ethnic groups (Table 3).

**Table 3:** **Regressionkoefficient, mmHg** (***p***-**value) in univariate and multivariate regressionanalyses adjusted for additional confounders**.

	**1: Ethnicity *N* = 92 (univariate)**	**2: 1 + Sodium reduction**	**3: 2 + Baseline blood pressure**	**4: 3 + Age**	**5: 4 + Duration**	**6: 5 + Female%**
Effect SBP,	1.28 (0.039)	1.32 (0.032)	1.15 (0.026)	1.08 (0.032)	1.12 (0.03)	1.07 (0.043)
Effect DBP,	0.63 (0.083)	0.60 (0.10)	0.50 (0.14)	0.46 (0.17)	0.55 (0.11)	0.61 (0.077)

In another additional analysis we excluded 35 study populations of Whites with a high sodium reduction to adjust the quantity of sodium reduction in Blacks and Whites (Table 2, column 5). This only changed the outcome blood pressures marginally (Figure 1, lines 7 and 14).

## Discussion

The present study, which attempted to adjust for baseline blood pressure and the quantity of sodium reduction showed that Asians had a non-significant trend toward a higher BP response to SR than whites, but they were also older, had a non-significantly higher mean baseline BP and a significantly higher baseline sodium intake (Table 2). Blacks had a non-significant trend toward a higher SBP response and a significant trend toward a higher DBP response to SR than Whites while matching the Whites on other baseline variables except quantity of SR (Table 2). The difference was unchanged after adjusting for quantity of SR (Table 2). The supplementary meta-regression analyses adjusted for confounders confirmed that the ethnic differences in blood pressure response to sodium reduction were small, although marginally significant for SBP. Thus there may be an unexplained additional effect of SR on BP, especially in Blacks, which however is small compared to previous unadjusted findings (Graudal et al., 2011). One explanation could be that there are few studies in Asians and Blacks. This might increase the risk of publication bias as suggested by our previous cumulative meta-analysis (Graudal et al., 1998), which showed a higher effect of SR on BP after publication of the first 7–8 studies, whereas a smaller and more stable effect was manifest after the publication of about 15 studies. This assumption is also indicated by the fact that the comparisons of Asian, Black, and White study populations from identical studies showed no statistical differences between the ethnic groups (Figure 1). The heterogeneity between the baseline characteristics was large and therefore it was difficult to adjust all 3 study-groups to identical baseline values, because adjustment of one baseline value created another imbalance between other baseline variables. However, in spite of these variations, the differences in effects of SR on BP between the ethnic groups were small.

Our results are in accordance with a recent co-operation between Cuban, Canadian, and American researchers, who compared Black and White Cubans living under similar socio-economic conditions and found that “skin color was unrelated to mean blood pressure or hypertensive status” (Ordúñez1 et al., 2013). The background for this study was the assumption by many scientists that the excess burden of hypertension among blacks was an inevitable phenomenon. However, the authors concluded that social conditions rather than ethnic group may determine the general development of excess hypertension in Blacks (Ordúñez1 et al., 2013). In that context it should be emphasized that the sodium reduction RCTs of Whites generally are performed in Europeans and Americans, whereas the RCTs of Blacks are performed in Black Americans and Africans, who socioeconomically are not comparable to White Americans and Europeans. In that context it is interesting that after adjustment for important confounders the difference between Whites and Blacks was small. If we had been able to adjust for socio-economic differences this last small difference in effect on DBP might have disappeared.

The possibility that socioeconomic conditions has an important influence on BP may also be reflected in the BP development in the United States during the 20th century. Not only did BP fall markedly in each new 10–year birth cohort from 1887 to 1975, the slope of the BP increase with age of each of these cohorts also decreased (Goff et al., 2001). The total fall in BP during the 20th century was dramatic and cannot solely be explained by the introduction of antihypertensive treatments, low-fat diets or decrease in the use of tobacco, as the BP fall started long before these interventions. The enormous socio-economic development in the United States is a much more likely explanation. Recently the fall in BP seems to have stopped, maybe due to the present overweight epidemic (Goff et al., 2012). In the Cuban study the mean SBP was about 120 mmHg (adults > 15 years) and the percentage of hypertension was about 31% in both Blacks and Whites. In US the mean SBP was about 127 mmHg for Blacks and about 122 mmHg for Whites (adults > 18 years) (Wright et al., 2011). The percentage of hypertension is about 37% for Blacks and 30% for Whites (Wright et al., 2011).

An IOM report from 2004 recommended that all African Americans eat less than 1500 mg of sodium, whereas whites could eat up to 2300 mg of sodium (Institute of Medicine, 2004). In contrast the recent IOM report from 2013 (Institute of Medicine, 2010) concluded “Given this background, overall, the committee found that the available evidence on associations between sodium intake and direct health outcomes is consistent with population-based efforts to lower excessive dietary sodium intakes, but it is not consistent with recommendations that encourage lowering of dietary sodium in the general population to 1500 mg per day. Further, as noted in the 2010 DGAC report, population subgroups, including those with diabetes, CKD, or preexisting CVD, individuals with hypertension, pre-hypertension, persons 51 years of age and older, and African Americans represent, in aggregate, a majority of the general U.S. population. Thus, when considered in light of the current state of the evidence on associations between sodium intake and direct health outcomes for these subgroups, except when data specifically indicate they are different, there is not sufficient evidence to support treating them differently from the general U.S. population.” The present study is in accordance with the 2013 IOM conclusion indicating no true ethnic dependent sensitivity to sodium.

In conclusion, ethnic differences in blood pressure response to sodium reduction seem smaller than previously observed. When the effect of SR on BP was adjusted for baseline blood pressure and quantity of sodium reduction there was a small statistically significant ethnic difference in SBP response and DBP response depending on the statistical method used, but the comparisons of Asian, Black and White study populations from studies investigating ethnic groups in identical studies showed no statistical differences in effect of SR on SBP or DBP between the ethnic groups in accordance with the conclusion of the 2013 IOM report. Whether the small observed differences in the present study are due to socio-economic factors or genetic factors remains to be setteled. Public guidelines like the American Guidelines (U.S. Department of Agriculture and U.S. Department of Health and Human Services 2010) might be changed to recommend the same levels of sodium intake irrespective of ethnicity.

### Conflict of interest statement

The authors declare that the research was conducted in the absence of any commercial or financial relationships that could be construed as a potential conflict of interest.
